# Endovascular repair of bilateral sciatic artery aneurysm with Covera® self expandable covered stents - case report

**DOI:** 10.1590/1677-5449.202200642

**Published:** 2023-06-30

**Authors:** Luisa Silveira Birck, Rodrigo Damazzini, Wiliam Perdomo Nunes, Thiago Filomena Lombard, Joel Alex Longhi

**Affiliations:** 1 Instituto de Saúde São Lucas de Pato Branco - ISSAL, Pato Branco, PR, Brasil.; 2 Grupo Hospitalar Conceição - GHC, Porto Alegre, RS, Brasil.

**Keywords:** aneurysm, sciatica, endovascular procedures, self expandable metal stent, endoprosthesis

## Abstract

A persistent sciatic artery is an embryological remnant of the internal iliac artery that occurs in 0.03% to 0.06% of the population and may develop aneurysmal degeneration. Aneurysms can lead to distal embolization with increased risk of limb loss, especially if the sciatic artery is the main arterial supply to the limb. A sciatic artery aneurysm must be treated whenever diagnosed, because of the high risk of complications. Treatment options include open, endovascular, or hybrid repair. This manuscript describes a patient with bilateral persistence of the sciatic arteries, both with aneurysmal degeneration, who underwent endovascular repair with Covera^®^ (Bard Medical, Georgia-USA) covered stents.

## INTRODUCTION

Persistent sciatic arteries are embryological remnants of the internal iliac artery, occurring in 0.03 to 0.06% of the population.^1-4^ They can be classified into two types: complete (more common, accounting for more than 80% of cases), in which the sciatic artery is the main artery responsible for the lower limb blood supply, or incomplete, in which the femoral artery is the predominant artery.^5,6^

Symptoms occur in more than 60% of cases^1,2^ and are generally related to the effects of compression of the sciatic nerve and aneurysmal degeneration. Presence of an aneurysm can lead to formation of mural thrombi with consequent distal embolization, provoking acute or chronic ischemia with increased risk of limb loss, primarily if the sciatic artery is the main arterial blood supply, as in the complete type of sciatic artery.

Treatment is indicated whenever a sciatic artery aneurysm is diagnosed, because of the high risk of complications.^3^ Treatment options include open, endovascular, or hybrid repair. The number of reports of endovascular treatment using covered stents has been increasing because of the high complexity of open repair, the increased likelihood of complications involving nerve damage, and the slow postoperative recovery.

This article will describe the case of a patient with complete persistent sciatic arteries bilaterally and aneurysmal degeneration of both arteries, repaired with Covera^®^ self-expanding covered stents (Bard Medical, Georgia, United States).

The study was approved by the institutional Ethics Committee (decision number 5.388.096; Ethics Appraisal Submission Certificate: 58110022.9.0000.5327).

## CASE REPORT

**Figure 1:**
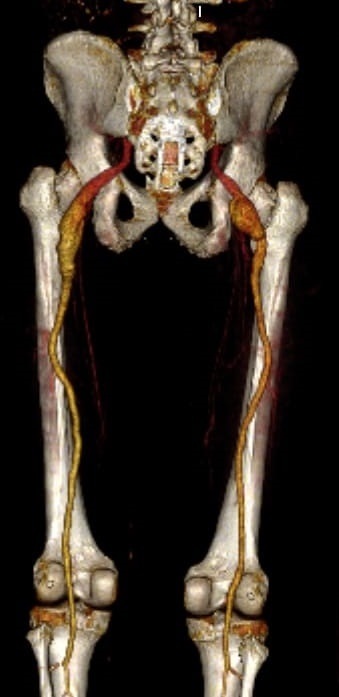
Three-dimensional (3D) angiotomography image showing bilateral sciatic artery aneurysms.

The patient was a 67-year-old white male with no known comorbidities and no history of smoking. He was treated at a hospital emergency department for an acute arterial occlusion of the femoropopliteal segment in the right lower limb, with successful thromboembolectomy via the popliteal artery. Later, angiotomography (angioCT) was performed to investigate the emboligenic agent, detecting complete persistent sciatic arteries with fusiform aneurysmal degeneration bilaterally (Figure 1). The aneurysm of the right sciatic artery was around 17 cm in length, with a maximum diameter of around 4 cm, with abundant mural thrombi, while the aneurysm on the left side was around 14 cm in length, with a maximum diameter of around 2.7 cm.

The patient complained of pain in the gluteal region, irradiating to the lower limbs, primarily when spending long periods in a sitting position. On physical examination, femoral pulses were weak on palpation bilaterally, but popliteal and distal pulses were normal.

The decision was taken to conduct bilateral repair, in view of the embolization and diameter of the aneurysms. Endovascular treatment was performed with Covera® self-expanding covered stents (Bard Medical, Georgia, United States), on the right side first (the symptomatic limb), using two 10 x 100 mm stents. Thirty days later, the aneurysm in the left lower limb was repaired using two stents: a 10 x 80 mm stent proximally and a 9 x 100 mm stent distally. The popliteal artery was dissected via a posterior access, with the patient in ventral decubitus, inserting a 9 F introducer sheath via retrograde puncture. After removal of the sheath, primary arteriorrhaphy was performed. There were no intercurrent conditions during the procedure. After hospital discharge, the patient was maintained on anticoagulation with rivaroxaban 20 mg/day and antiplatelet aggregation with acetylsalicylic acid (ASA) 100 mg/day.

At a follow-up consultation 15 days after the procedure, the patient reported gluteal pain, more intense on the left, but symptoms exhibited progressive improvement with dipyrone, tramadol, and pregabalin. He showed no signs or symptoms of limb ischemia, with popliteal and distal pulses still normal on palpation, and there were also no changes related to sensory or motor nerve.

**Figure 2:**
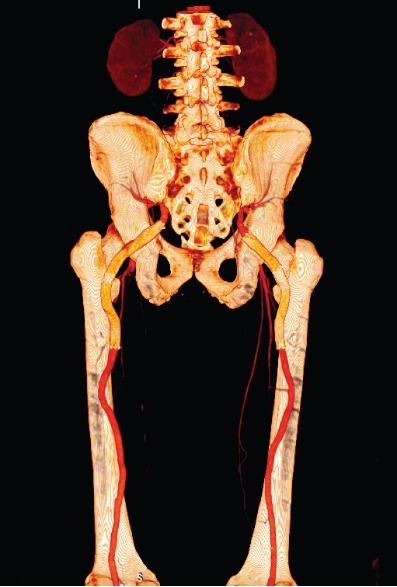
Three-dimensional (3D) angiotomography control image 30 days after repair of bilateral sciatic artery aneurysms (coronal plane). Patent stents free from endoleaks.

**Figure 3:**
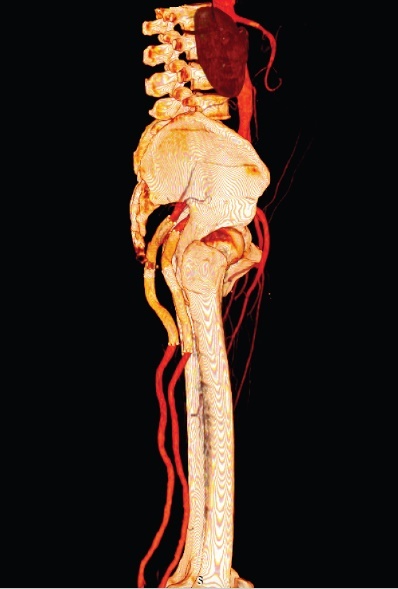
Three-dimensional (3D) angiotomography control image 30 days after repair of bilateral sciatic artery aneurysms (sagittal plane).

After 30 days, control angioCT was performed, demonstrating patent stents free from endoleaks (Figures 2 and 3). Around 9 months after treatment, the patient attended a follow-up consultation, where he was asymptomatic, with popliteal and distal pulses palpable bilaterally, and free from signs of limb ischemia. Another angioCT was ordered for follow-up, with no result to date.

## DISCUSSION

The sciatic artery is an embryonic vessel that normally regresses, forming the proximal part of the inferior gluteal artery after the third month of intrauterine life. Persistence of the sciatic artery is a rare anomaly of development, in which the internal iliac artery and the embryonic axial artery continue to provide the blood supply to the lower limb after birth.^3,7^ They can be classified as one of two types: complete (more common, observed in more than 80% of cases), in which the popliteal artery primarily receives blood flow from the sciatic artery and the superficial femoral artery is hypoplastic or absent; or incomplete, in which the arterial flow to the extremity predominantly derives from the femoropopliteal axis.^1^ The condition affects both sexes equally^3,8^ and can occur bilaterally in up to 50% of cases.^8^

Persistence of the sciatic artery can be symptomatic.^1,2^ Mean age at presentation of symptoms is from 40 to 50 years, and manifestations are generally related to the effects of compression of the sciatic nerve and aneurysmal degeneration.^3,9^ In the present case, the patient had the complete type, bilaterally, presenting with symptoms of sciatic nerve compression and aneurysmal degeneration with embolization and acute ischemia of the right lower limb.

The sciatic artery has a predisposition to premature atherosclerosis because of connective tissue hypoplasia, leading to aneurysms and thromboembolic events.^8^ Aneurysmal degeneration is described in up to 60% of cases,^1^ which may be the result of repeated traumatisms. If the femoral artery is hypoplastic or absent, the patient will have palpable popliteal and distal pulses, but the femoral pulse will be diminished or absent - known as Cowie’s sign, which is strongly suggestive of the complete presentation.^10^ Aneurysm rupture is also described, although it occurs rarely.^3^

Three main treatments for sciatic artery aneurysms are described in the literature: open repair, coil embolization (in incomplete presentations), and endovascular exclusion of the aneurysm with deployment of covered stents (complete presentations).^1^ In the present case, bilateral aneurysm repair using two overlapping covered stents on each side was chosen, because of the high complexity of open repair.

A systematic review from 2020 (Charisis et al.^1^ ) analyzed 15 studies of treatment of sciatic artery aneurysms. All of the patients underwent endovascular repair with covered stents, using a median of 1.5 stents per aneurysm. Endovascular treatment was successful in all cases, with a very low rate of periprocedural complications. Over a mean follow-up of 14 months, the aneurysms remained excluded and all patients were free from symptoms. At follow-up, 15 days after the procedure, the patient in the present study had moderate gluteal pain, primarily on the left side, probably because of thrombosis of the aneurysm sac and exclusion of vessels feeding the sciatic nerve. The left side did not have significant mural thrombi, which may be the reason why pain was more intense on the left.

With relation to the route of access, the access chosen in the present case was retrograde via dissection of the popliteal artery. Studies propose puncture of the ipsilateral popliteal and a retrograde approach because of the possible difficulty of deploying a long stent via an anterograde approach through tortuous vessels.^11,12^ In another systematic review, 70% of interventions were via a retrograde approach and 30% were anterograde.^1^

In a review by Charisis et al.,^1^ Viabahn^®^ covered stents (W. L. Gore and Associates, Flagstaff, Arizona, United States) were most often used to repair aneurysms of the sciatic artery, while there were also descriptions of use of the iliac extension for the Excluder^®^ endoprosthesis, Hemobahn stents^®^ (W. L. Gore and Associates, Flagstaff, Arizona, United States), and Wallgraft stents^®^ (Boston Scientific, Marlborough, Massachusetts, United States). However, the only stents with the flexibility and radial strength characteristics necessary for the case that were available at our institution for treatment on the Unified Health System (SUS - Sistema Único de Saúde) were Covera^®^ covered stents (Bard Medical, Georgia, United States).

The Covera^®^ stent is a flexible, self-expanding, covered stent made from a nitinol structure encapsulated in expanded polytetrafluoroethylene (ePTFE). It has a helicoidal design for radial resistance and flexibility, and was developed for flexion, compression, and torsion with helical struts and angled bridges. It is officially intended to be used to treat stenosis of the arteriovenous anastomoses of hemodialysis fistulas. However, it has also been described as a possible option for constructing chimney grafts to repair juxtarenal abdominal aortic aneurysms, used as a bridging stent to reline renal and visceral arteries in endovascular repair of the thoracoabdominal aortic aneurysms and has recently been indicated for treatment of atherosclerotic lesions of the iliac artery.^13,14^ To date, no cases have been found in the literature of use of Covera^®^ stents to treat sciatic artery aneurysm.

There is no consensus on maintenance of anticoagulation or platelet antiaggregation after treatment of sciatic artery aneurysms with covered stents, but in the present case, it was decided to maintain rivaroxaban and ASA, since the stent sites would be permanently subjected to traumatic stresses and traction forces and the patient had a low risk of hemorrhagic events.

Although surgical repair of sciatic artery aneurysms is considered a well-established method, there is no evidence that it is superior to endovascular surgery,^1,15^ since data are scarce and the prevalence of the disease is low. The technical complexity and invasive nature of surgery and the greater length of hospital stay, in conjunction with the new technological advances in endovascular treatment have led to a growing number of patients being treated with covered stents,^9,15^ since the procedure is minimally invasive, involves low risk of sciatic nerve damage, and still leaves the option of femoropopliteal or iliac-popliteal bypass in reserve in case of treatment failure.

It can be concluded, on the basis of this report, that endovascular repair of bilateral sciatic artery aneurysms was feasible using Covera^®^ covered stents, with no periprocedural complications and evidence of stent patency at 9 months’ follow-up. One limitation of this study is the lack of long-term follow-up imaging exams.
