# Supplementary material for: Endovascular repair of bilateral sciatic artery aneurysm with Covera® self expandable covered stents - case report
Source: J Vasc Bras. 2023 Jun 30;22:e20220064. doi: 10.1590/1677-5449.202200642 (PMC10421579; doi:10.1590/1677-5449.202200642)
Supplement: Translation

# Translation: Tratamento endovascular de aneurisma de artéria isquiática bilateral com stent recoberto autoexpansível Covera^®^ - relato de caso

**DOI:** 10.1590/1677-5449.202200641

**Authors:** Luisa Silveira Birck, Rodrigo Damazzini, Wiliam Perdomo Nunes, Thiago Filomena Lombard, Joel Alex Longhi

**Affiliations:** 1 Instituto de Saúde São Lucas de Pato Branco - ISSAL, Pato Branco, PR, Brasil.; 2 Grupo Hospitalar Conceição - GHC, Porto Alegre, RS, Brasil.

**Keywords:** aneurisma, isquiática, procedimentos endovasculares, stents metálicos autoexpansíveis, endopróteses

## Abstract

A persistência da artéria isquiática é um remanescente embriológico da artéria ilíaca interna que ocorre em 0,03% a 0,06% da população, podendo evoluir com degeneração aneurismática. A presença do aneurisma pode levar a embolização, com aumento de risco de perda do membro, principalmente se a artéria isquiática for seu principal suprimento arterial. O tratamento do aneurisma de artéria isquiática está indicado sempre que diagnosticado, devido ao alto risco de complicações. Entre as opções de tratamento, estão o tratamento aberto convencional, o tratamento endovascular e o tratamento híbrido. No presente estudo, será descrito o caso de um paciente apresentando persistência completa das artérias isquiáticas bilateralmente, com degeneração aneurismática de ambas, corrigida de forma endovascular com stent recoberto Covera^®^ (Bard Medical, Geórgia, Estados Unidos).

## INTRODUÇÃO

A persistência da artéria isquiática é um remanescente embriológico da artéria ilíaca interna que ocorre em 0,03% a 0,06% da população^1-4^ . Pode ser dividida em dois tipos: o completo (mais comum, ocorrendo em mais de 80% dos casos), em que a artéria isquiática é a principal responsável pelo suprimento sanguíneo do membro inferior, e o incompleto, no qual a artéria femoral é predominante^5,6^ .

Sintomas ocorrem em mais de 60% dos casos^1,2^ , geralmente relacionados a efeitos de compressão do nervo ciático e degeneração aneurismática. A presença do aneurisma pode levar à formação de trombos murais com consequente embolização distal, provocando isquemia aguda ou crônica com aumento de risco de perda do membro, principalmente se a artéria isquiática for o principal suprimento arterial, como no tipo completo.

O tratamento do aneurisma de artéria isquiática está indicado sempre que diagnosticado, devido ao alto risco de complicações^3^ . Entre as opções de tratamento, estão o reparo aberto, endovascular ou híbrido. Atualmente tem aumentado o número de relatos de tratamento endovascular com utilização de stents recobertos, devido à alta complexidade do reparo aberto, à maior possibilidade de complicações como lesão nervosa e à lenta recuperação pós-operatória.

No presente estudo, será descrito o caso de um paciente apresentando persistência completa das artérias isquiáticas bilateralmente, com degeneração aneurismática de ambas, corrigida com stent recoberto autoexpansível Covera^®^ (Bard Medical, Geórgia, Estados Unidos).

O protocolo foi aprovado pelo Comitê de Ética de nossa instituição (parecer número 5.388.096; Certificado de Apresentação de Apreciação Ética: 58110022.9.0000.5327).

## RELATO DE CASO

O paciente era do sexo masculino, branco, com 67 anos, sem comorbidades conhecidas e sem histórico de tabagismo. Foi atendido na emergência hospitalar devido a um quadro de oclusão arterial aguda do segmento femoropoplíteo no membro inferior direito, sendo realizada tromboembolectomia bem-sucedida através da artéria poplítea. Posteriormente, foi realizada angiotomografia (angioTC) para investigação do foco emboligênico, na qual foi detectada persistência de artéria isquiática do tipo completo com degeneração aneurismática fusiforme bilateralmente (Figura 1). O aneurisma da artéria isquiática direita media cerca de 17 cm de extensão, com maior diâmetro de cerca de 4 cm, apresentando trombos murais exuberantes, e à esquerda apresentava extensão de cerca de 14 cm, com maior diâmetro de cerca de 2,7 cm.

**Figura 1:** Imagem tridimensional (3D) da angiotomografia demonstrando aneurisma em artérias isquiáticas bilateralmente.

O paciente apresentava dor em região glútea com irradiação para membros inferiores, principalmente ao permanecer longos períodos sentado. Ao exame físico, os pulsos femorais eram reduzidos à palpação bilateralmente, porém os pulsos poplíteos e distais apresentavam-se normais.

Devido ao quadro de embolização e ao diâmetro dos aneurismas, optou-se pela correção bilateral. Foi realizado tratamento endovascular com stent recoberto autoexpansível Covera^®^ (Bard Medical, Geórgia, Estados Unidos), primeiramente à direita (membro sintomático), com dois stents 10 x 100 mm. Trinta dias após, foi corrigido o aneurisma no membro inferior esquerdo, com dois stents: 10 x 80 mm proximal e 9 x 100 mm distal. Realizou-se a dissecção da artéria poplítea através de acesso posterior, com o paciente em decúbito ventral, sendo puncionada bainha introdutora 9 F via retrógrada. Posteriormente à retirada da bainha, foi confeccionada rafia arterial primária. Não houve intercorrências durante o procedimento. Optou-se por manter, após a alta hospitalar, anticoagulação com rivaroxaban 20 mg/dia e antiagregação plaquetária com ácido acetilsalicílico (AAS) 100 mg/dia.

No seguimento após 15 dias do procedimento, o paciente relatou dor em glúteos mais intensa à esquerda, apresentando melhora sintomática progressiva com o uso dipirona, tramadol e pregabalina. Não apresentou sinais e sintomas de isquemia de membros, permanecendo os pulsos poplíteos e distais normais à palpação, e também não houve quadro de alterações neurológicas sensitivas ou motoras.

Após 30 dias, realizou angioTC de controle, demonstrando os stents pérvios e sem *endoleaks* (Figuras 2 e 3). Cerca de 9 meses após o tratamento, o paciente compareceu em consulta de retorno, mantendo-se assintomático, com pulsos poplíteos e distais palpáveis bilateralmente e sem sinais de isquemia de membros. Foi solicitada nova angioTC para seguimento, ainda sem resultado até o presente momento.

**Figura 2:** Imagem tridimensional (3D) de angiotomografia de controle após 30 dias da correção de aneurismas de artérias isquiáticas bilateralmente (corte coronal). Stents pérvios e sem *endoleaks*.

**Figura 3:** Imagem tridimensional (3D) de angiotomografia de controle após 30 dias da correção de aneurismas de artérias isquiáticas bilateralmente (corte sagital). Stents pérvios e sem *endoleaks*.

## DISCUSSÃO

A artéria isquiática é um vaso embrionário que normalmente regride, formando a parte proximal da artéria glútea inferior após o terceiro mês de vida intrauterina. A persistência da artéria isquiática é uma rara anomalia do desenvolvimento, na qual a artéria ilíaca interna e a artéria axial embrionária continuam a fornecer suprimento sanguíneo para o membro inferior após o nascimento^3,7^ . Pode ser classificada em dois tipos: tipo completo (mais comum, observado em mais de 80% dos casos), no qual a artéria poplítea recebe fluxo sanguíneo principalmente da artéria isquiática, com a artéria femoral superficial se apresentando hipoplásica ou ausente; e o tipo incompleto, em que o fluxo arterial para a extremidade provém predominantemente do eixo femoropoplíteo^1^ . Afeta igualmente ambos os sexos^3,8^ e pode ocorrer bilateralmente em até 50% dos casos^8^ .

A persistência da artéria isquiática pode ser sintomática^1,2^ . A idade média de apresentação dos sintomas está entre os 40 e 50 anos, geralmente relacionados a efeitos de compressão do nervo ciático e degeneração aneurismática^3,9^ . No presente caso, o paciente exibia o tipo completo, com acometimento bilateral, apresentando-se com sintomas de compressão do nervo ciático e degeneração aneurismática com embolização e isquemia aguda do membro inferior direito.

Devido à hipoplasia do tecido conjuntivo da artéria isquiática, esta mostra maior predisposição à aterosclerose precoce, levando a aneurismas e eventos tromboembólicos^8^ . É descrita degeneração aneurismática em até 60% dos casos^1^ , o que pode ser resultado de traumatismos repetidos. Se a artéria femoral estiver hipoplásica ou ausente, o paciente terá pulsos poplíteo e distais palpáveis e pulso femoral reduzido ou abolido - sinal de Cowie -, sendo fortemente sugestivos de apresentação completa^10^ . Também é descrita a ruptura do aneurisma, ocorrendo raramente^3^ .

Conforme a literatura, três principais tratamentos de aneurismas de artérias isquiáticas foram relatados, incluindo reparo aberto, embolização com mola (tipos incompletos) e exclusão endovascular de aneurisma com implante de stent recoberto (tipos completos)^1^ . No presente estudo, foi optado pelo tratamento com stent recoberto dos aneurismas bilateralmente, devido à alta complexidade do reparo aberto neste caso, sendo utilizados dois stents recobertos sobrepostos em cada lado.

Em uma revisão sistemática de 2020 (Charisis et al.^1^ ), 15 estudos sobre tratamento de aneurismas de artéria isquiática foram analisados. Todos os pacientes foram submetidos ao reparo endovascular com stents recobertos, sendo utilizada uma mediana de 1,5 stents para cada lesão. A terapia endovascular foi bem-sucedida em todos os casos, com uma taxa de complicação periprocedimento muito baixa. Durante um seguimento médio de 14 meses, os aneurismas permaneceram obliterados, e todos os pacientes estavam livres de sintomas. O paciente do presente estudo apresentou dor glútea moderada principalmente do lado esquerdo no seguimento após 15 dias do procedimento, provavelmente por trombose do saco aneurismático e exclusão de vasos nutridores do nervo ciático. O lado esquerdo não apresentava trombos murais significativos, podendo ser o motivo pelo qual apresentou dor mais intensa à esquerda.

Em relação à via de acesso, no presente estudo foi optado pela via retrógrada através da dissecção da artéria poplítea. Estudos propuseram a punção poplítea ipsilateral e abordagem retrógrada devido a possíveis dificuldades na implantação de um stent longo por via anterógrada através de vasos tortuosos^11,12^ . Em outra revisão sistemática, foram realizadas 70% das intervenções por via retrógrada e 30% por via anterógrada^1^ .

Na revisão de Charisis et al.^1^ , houve a utilização mais frequente do stent recoberto Viabahn^®^ (W. L. Gore and Associates, Flagstaff, Arizona, Estados Unidos) para o reparo do aneurisma de artéria isquiática e também foi descrito o uso de extensão ilíaca de endoprótese Excluder^®^, além de stents Hemobahn^®^ (W. L. Gore and Associates, Flagstaff, Arizona, Estados Unidos) e Wallgraft^®^ (Boston Scientific, Marlborough, Massachusetts, Estados Unidos). Porém, somente havia disponível em nossa instituição para tratamento pelo Sistema Único de Saúde o stent recoberto Covera^®^ (Bard Medical, Geórgia, Estados Unidos), com características adequadas de flexibilidade e força radial necessárias para o caso.

O stent Covera^®^ é um stent recoberto flexível e autoexpansível, composto por politetrafluoretileno expandido (ePTFE) encapsulando uma estrutura de nitinol. Apresenta um desenho helicoidal para resistência radial e flexibilidade, projetado para flexão, compressão e torção com escoras helicoidais e pontes anguladas. Foi oficialmente destinado a ser utilizado no tratamento de estenoses de anastomoses arteriovenosas de fístulas para hemodiálise. No entanto, também tem sido relatado como possível alternativa para confecção de chaminés no reparo de aneurismas de aorta abdominal justarrenal, utilizado como stent-ponte para recobrir artérias renais e viscerais na correção endovascular de aneurismas da aorta toraco-abdominal, e recentemente foi indicado para o tratamento de lesões ateroscleróticas da artéria ilíaca^13,14^ . Não foi encontrado na literatura o uso do stent Covera^®^ para tratamento de aneurisma de artéria isquiática até o presente momento.

Não há um consenso sobre manter anticoagulação ou antiagregação plaquetária após o tratamento dos aneurismas de artéria isquiática com stents recobertos, porém, no presente caso, foi optado por manter rivaroxaban e AAS, já que o local da implantação dos stents será permanentemente submetido a estresses traumáticos e forças de tração e o paciente apresenta baixo risco para eventos hemorrágicos.

Embora a correção cirúrgica dos aneurismas de artéria isquiática seja considerada uma abordagem bem estabelecida, não há evidências de superioridade da cirurgia à terapia endovascular^1,15^ devido aos dados escassos e à baixa prevalência da doença. A complexidade técnica e a natureza invasiva da cirurgia, associadas ao maior tempo de internação e aos novos avanços tecnológicos da terapia endovascular, levaram a um número crescente de pacientes submetidos ao tratamento com stent recoberto^9,15^ , já que é um procedimento minimamente invasivo associado a baixo risco de lesão do nervo ciático, preservando a opção de derivação femoropoplítea ou ilíaco-poplítea em caso de falha.

Pode-se concluir, a partir deste relato, que o reparo endovascular de aneurisma bilateral da artéria isquiática utilizando stent recoberto Covera^®^ foi exequível, sem complicações periprocedimento e com sinais de perviedade dos stents com 9 meses de acompanhamento. Este estudo apresenta como limitação o fato de não haver exame de imagem de seguimento a longo prazo.
